# Clinical characteristics and comparison of the outcome in young versus older patients with upper gastrointestinal carcinoma

**DOI:** 10.1007/s00432-020-03302-x

**Published:** 2020-07-02

**Authors:** Hannah Christina Puhr, Alexander Karner, Hossein Taghizadeh, Gerd Jomrich, Sebastian Friedrich Schoppmann, Matthias Preusser, Aysegül Ilhan-Mutlu

**Affiliations:** 1 Division of Oncology, Department of Medicine I, Medical University of Vienna, Waehringer Guertel 18-20, 1090 Vienna, Austria; 2 Department of Surgery, Medical University of Vienna, Waehringer Guertel 18-20, 1090 Vienna, Austria; 3 Upper-GI Tumours Unit, Comprehensive Cancer Centre Vienna, Medical University of Vienna, Vienna, Austria

**Keywords:** Gastroesophageal tumour, Oesophageal tumour, Gastroesophageal junction tumour, Gastric cancer, Young, Old

## Abstract

**Background:**

The clinical behaviour and outcome of young patients with gastroesophageal tumours (GET) is surmised to differ from older patients, yet data on the comparison of these two patient subgroups is scarce. This study focuses on the investigation of the clinical characteristics and survival outcome of younger-age people with GET, when compared to older patients.

**Methods:**

Patients diagnosed with GET at the Medical University of Vienna between 2004 and 2016 were included in this study. Clinical parameters and the overall survival (OS) were compared between young (≤ 45 years) and elderly (≥ 65 years) patients.

**Results:**

Among 796 patients, who were eligible for this analysis, fifty-eight patients (7%) were ≤ 45 years at the initial onset of the disease. These 58 young patients were then matched to elderly patients based on the gender, tumour stage, histology and tumour location. The number of metastatic lesions was significantly higher among young patients (*p* < 0.05). In a non-metastatic setting younger patients showed a significant longer OS than older patients (median 1226 versus 801 days, *p* = 0.028). Furthermore, young patients with extensive metastatic disease (2 or more metastatic site) had a significantly poorer OS than elderly patients (median 450 versus 646 days, *p* = 0.033).

**Conclusion:**

These results indicate that young patients might be diagnosed very late, which might lead to the development of a more aggressive disease compared to older patients, but a relatively long OS when diagnosed and treated in a non-metastatic setting. Thus, screening methods for younger patients might be considerable to enhance the outcome of young patients with GET.

## Background

Gastroesophageal tumours (GET), including stomach, gastroesophageal junction and oesophagus, is the fourth most common cancer, and the second leading cause of cancer-related deaths worldwide (Smyth et al. 2016). The incidence of GET is decreasing in Western countries, whereas it remains high in Asia (Van Cutsem et al. 2016). GET is considered to be the disease of the elderly and its prevalence increases with age (Anderson et al. 2010). The mean age of GET is over fifty years (Van Cutsem et al. 2016), but there are some proportions of patients who are diagnosed with gastric cancer at younger ages. The definition of young age of GET is varying, as some literature identifies forty or forty-five years and below. Approximately 10% of young GET patients have a positive family history (Kokkola and Sipponen 2001).

There is varying information on the incidence, clinicopathological characteristics and outcome of young-age GET patients. The incidence of young age GET ranges between 2 and 15% (Carvalho et al. 2004; Llanos et al. 2006; Santoro et al. 2007). Some reports showed advanced lesions at presentation with higher proportions of undifferentiated (Lai et al. 2008) and biologically more aggressive tumours (Saito et al. 2012). GET are found predominantly in men; however, female patients represented more often at younger ages (Kong et al. 2012; Liu et al. 2016; Wang et al. 2016). The outcomes of these patients with GET are controversial, as previous studies demonstrated favourable, equivalent or even poorer prognoses when compared to older population (Kong et al. 2012; Smith and Stabile 2009; Takatsu et al. 2016; Theuer et al. 1996). The most important factor for the prognosis is the stage of the cancer at diagnosis. The majority of patients are diagnosed at an advanced stage and their survival is very poor.

GET is usually asymptomatic in early stages and symptoms such as weight loss, dysphagia and iron deficiency anaemia develop mostly in advanced tumour stages (Smyth et al. 2016). Overlooking of early symptoms is more common in younger patients as these symptoms are often considered to be in scope of a banal gastroenteritis. Early diagnosis of young GET patients is problematic, since routine screening investigations in many countries do not usually include people at younger ages. Thus, information of these young cancer patients, especially in early stages, is scarce.

In this retrospective monocentric survey, we focused on the investigation of the clinical characteristics and survival outcomes of GET patients in younger and older ages, who were diagnosed and treated at our clinic.

## Methods

### Patients’ collection

From 2004 to 2016, the medical records of the patients diagnosed and treated with gastroesophageal tumours at the General Hospital Vienna, Medical University of Vienna, Austria were retrospectively investigated.

Both patients with squamous cell carcinomas (SCC) and adenocarcinomas were included. Upper gastrointestinal cancer was defined as cancer located in the oesophagus, gastrointestinal junction and stomach.

All patients had a pathologically confirmed tumour specimen, either from biopsy or from surgical resection, reviewed by an experienced pathologist. Following clinical data were routinely collected and obtained from the patient database of the General Hospital Vienna, Austria: nicotine intake status, histopathological data of the tumour specimen including Her2 status, grading, staging, tumour location, metastatic status, treatment of the tumour (neoadjuvant treatment, surgical resection, adjuvant treatment or palliative treatment, administration of radiation therapy), laboratory findings of circulating tumour marker (carcinoembryonic antigen [CEA] and CA19-9 [carbohydrate antigen 19-9]) recurrence and survival outcomes. Men and women older than 18 years of age are included.

Young age was defined as ≤ 45, whereas old age was identified to be ≥ 65 years. Young and old patients were matched manually by exploring the collected data in a Microsoft Excel list. No specific software was used for the matching process. The patients were matchedbased on gender, location of the tumour (oesophagus/gastroesophageal junction/gastric), histology (adenocarcinoma/squamous cell carcinoma) and metastatic status at the initial presentation of the tumour. If a single young patient had potentially more than one old matching partner, the index patient was matched with an old patient having a similar survival time. This study was approved by the ethics committee of the Medical University of Vienna under the reference number 2267/2016.

### Response evaluation

Patients who were administered an anti-tumour treatment received a computed tomography every 3 months within the first year of the diagnosis. The amount of tumour shrinkage was investigated based on computed tomography images and the tumour response was classified in accordance with RECIST (Eisenhauer et al. 2009).

### Her2 analysis

Tumours were tested for Her2 status with immunohistochemistry (Hercep Test, Dako, Denmark) and fluorescence in-situ hybridization (FISH, Her2 IFSH pharmDx, Dako). For FISH, HER2 gene copy number and centromere enumerator probe 17 (Cep17) were investigated. The pathologists reported average copy numbers of Her2 and Cep17. The diagnosis criteria were based on Hofmann and colleagues (Hofmann et al. 2008). Patients were administered trastuzumab if their tumour samples were scored as 3 + on immunohistochemistry or in case of 2 +, if they were FISH positive (Her2:Cep17 ratio > 2).

### Statistical analysis

Patients without an event (death) were censored at the date that they were last known to be alive. Overall survival (OS) was calculated from the date of initial diagnosis of gastric cancer to the death of patient or patient’s last follow-up date. Kaplan–Meier survival estimates with log rank test and Cox regression analyses of OS were done. All reported values are two sided and *p* value was considered to be significant when < 0.05. Due to the hypothesis generating design of the current study no correction for multiple testing was applied.

## Results

### Patient´s characteristics

#### Entire cohort

In our institution, 885 patients were diagnosed with a GET between 2004 and 2016. 89 patients were presented at the outpatient clinic only once, where only sparse information of the disease course was available. Among 796 patients with sufficient hospital data, 58 (7%) were diagnosed with a GET before the age of 45. Among the young patients, the percentage of cases with metastatic disease at the initial disease presentation (stage IV) was slightly higher than the entire cohort, which however did not reach the statistical significance (25 patients (43%) in young group versus 274 patients (37%) within the rest; *p* = 0.4). Gender distribution had a tendency of having more female patients among the young group, which again was not statistically significant [19 female patients (33%) in young group versus 205 female patients within the rest of the cohort (28%); *p* = 0.4]. Younger patients had significantly less proportion of oesophageal carcinoma as location of the tumour when compared to other patients [9 patients with oesophageal carcinoma (15%) in young group versus 223 patients (30%) in other group; *p* < 0.001]. Most probably as a consequence of having fewer oesophageal cancer patients, younger patients had a slightly lower proportion of squamous cell carcinoma [6 patients with SCC (10%) among young patients versus 142 patients with SCC (19%) among older patients; *p* = 0.09].

#### Young versus old patients, selected group

The 58 patients, whose age was younger than 45, were matched with GET patients older than 65 based on the gender, tumour location, histology and metastatic status at the initial onset of the disease. Baseline characteristics of the patient population are described in Table 1. The median age was 41 years (ranging between 27 and 45 years) in the young group and 71 years (ranging between 65 and 83 years) in the old group. There were 19 female patients (32%) in both groups. Six patients (10% of the young group) were diagnosed with cancer at the age of under 30. The majority of the patient cohort composed of white Caucasian patients having only one patient with Asian origin in both groups. Nicotine consumption was observed in 24 (41%) and 19 (33%) patients in young and old group without any statistical significance (*p* = 0.59), respectively.

**Table 1:** Patients’ demographic and baseline characteristics

	Young	Old	Significance
No. of patients	58	58	
Age (years)	41	71	
Women	19	19	
Ethnic origin			
White	57	57	
Asian	1	1	
Black	0	0	
Nicotine abuse	24	19	n.s
Primary tumour site			
Stomach	33	33	
Oesophagus	9	9	
GEJ	16	16	
Histology			
Adenocarcinoma	52	52	
SCC	6	6	
Grading			
GII	15	21	n.s
GIII	36	31	
Initial tumour stage			
I	5	6	n.s
II	12	12	
III	16	12	
IV	25	25	
Metastatic sites per patient			
1	9	17	0.02
2 and more	16	8	
Location of metastasis			
Peritoneum	12	6	0.03
Liver	8	15	
Lymph nodes	12	5	
*H. Pylori* (yes)	10	11	n.s
Her2 (yes)	4	2	n.s

*GEJ* gastroesophageal junction, *Her2* human epidermal growth receptor 2, *H. pylori Helicobacter pylori*

The cases with the histology of adenocarcinoma and SCC were identical in both groups with 52 (90%) and 6 (10%) patients, respectively. In both groups, 25 patients (43%) had metastatic disease already at the initial presentation of the cancer. Younger patients had a slightly higher proportion of poorly differentiated tumours (GIII *n* = 36, 62%) compared to older patients (GIII *n* = 31, 53%) (*p* = 0.48). Among the young patients’ group, 9 (16%) and 16 (27%) patients presented with one and two metastatic sites at the initial presentation, respectively, whereas this was 17 (30%) and 8 (14%) among the older group (*p* = 0.02). Interestingly, young patients developed statistically more lymph node and peritoneum metastases, whereas older patients had a tendency to generate metastasis to the liver (*p* = 0.03). Helicobacter pylori and human epithelial growth factor receptor 2 (Her2) findings were similar in both patient groups.

The median observation time was 421 days (min 29 days, max 5131 days) in the young group and 523 days (min 50 days, max 4230 days) in the old group.

### Treatment modalities

Type of treatment and the type of chemotherapy regimen did not differ in both groups (Table 2). The median number of chemotherapy cycles within the palliative setting was 6 and 5 cycles in young and old groups, respectively, which was not statistically different (*p* = 0.5) Due to the retrospective nature of this study, not all side effects of the chemotherapy could be obtained from the hospital chart data. Based on the available data of the palliative chemotherapy setting, the most observed side effects were nausea, blood count changes and diarrhoea. The distribution of the side effects between young and old patients with upper GI-carcinoma was almost identical.

**Table 2:** Treatment modalities and side effects of the palliative chemotherapy

	Young	Old	*p*
**Treatment**			n.s
Primary resection	11	16	
With neoadjuvant CHT	22	13	
With adjuvant CHT	3	2	
Palliative CHT	20	21	
**Chemotherapy regimen**			
Neoadjuvant (yes)			
Cisplatin/Docetaxel	4	0	
DCF	2	1	
EOX	6	3	
Docetaxel	2	1	
Cisplatin/5-FU	4	5	
Others	1	4	
Palliative (yes)			
Average cycles of CHT in setting (median)palliative	6	5	n.s
TOGA	3	2	
DCF	9	6	
EOX	3	7	
Cisplatin/5-FU	0	2	
FOLFOX	3	4	
Cisplatin/Docetaxel	2	2	
Oxaliplatin/Docetaxel	2	1	
Others	6	3	
Xeloda	1	3	
XELOX	7	3	
**Side effects**			n.s
Nausea	7	6	
Mucositis	3	0	
Diarrhoea	4	2	
Blood count	4	6	
Polyneuropathy	1	2	
Acute kidney injury	0	2	
Fatigue	2	2	
Flush	2	0	

*CHT* chemotherapy, *DCF* docetaxel/cisplatin/5-fluoroucil, *EOX* epirubicin/oxaliplatin/xeloda, *5-FU* 5-fluoroucil, *TOGA* herceptin/cisplatin/5-fluoroucil, *FOLFOX* 5-fluoroucil/oxaliplatin, *XELOX* xeloda/oxaliplatin

### Survival outcomes

#### Overall survival compared between young and older patient cohorts

**Fig. 1:**
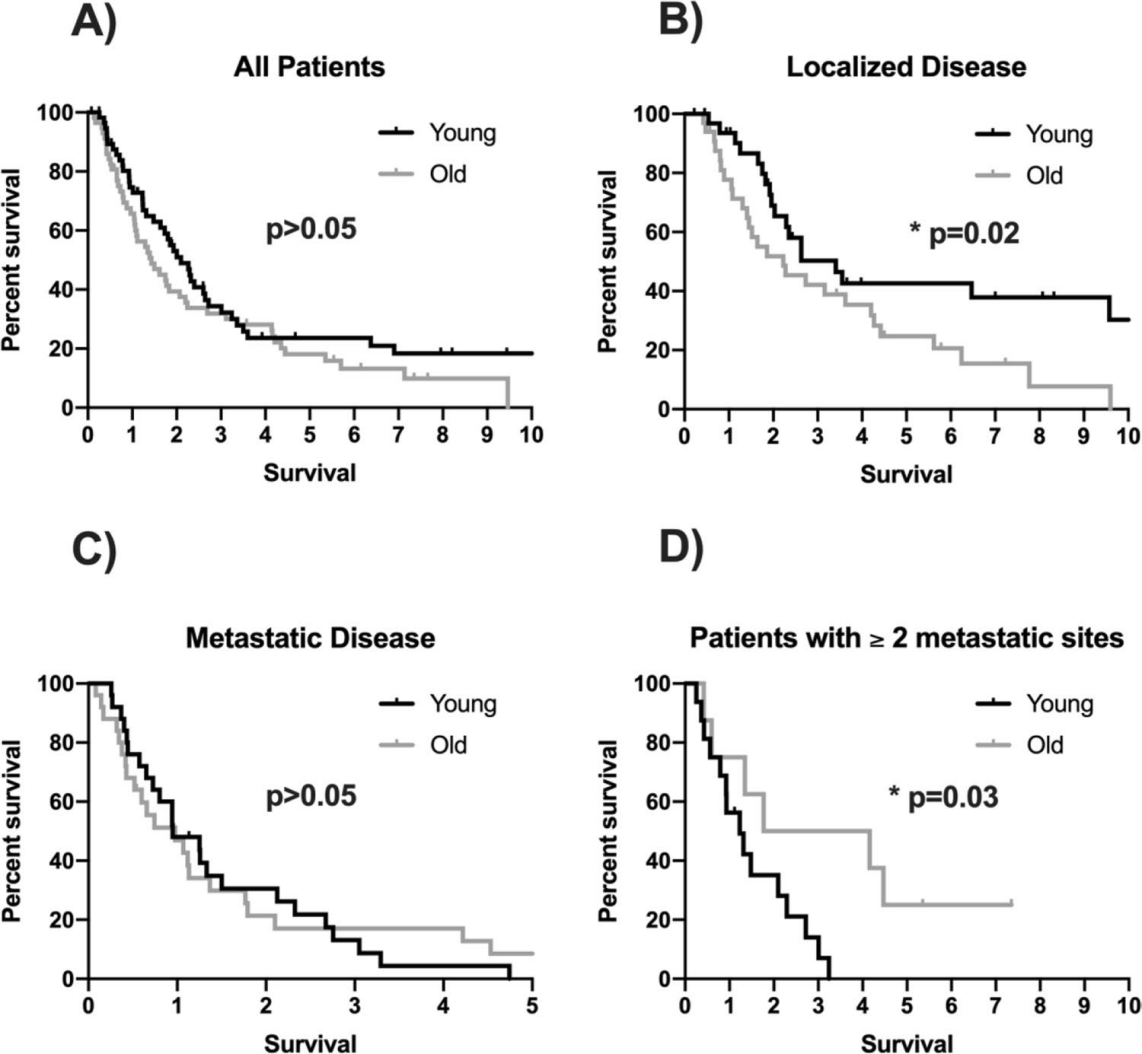
**a** Kaplan-Meier survival curve of the overall survival in patients with upper GI tumour in younger versus older age. **b** Kaplan-Meier survival curve of the overall survival in patients with upper GI tumour in younger versus older age in an initially non-metastatic setting. **c** Kaplan–Meier survival curve of the overall survival in patients with upper GI tumour in younger versus older age with an initial metastatic disease. **d** Kaplan–Meier survival curve of the overall survival in patients with upper GI tumour in younger versus older age in a setting with 2 or more metastatic sites

The overall survival (OS) did not differ statistical significantly in both groups when all patients of the young and old groups are included (median OS in younger cohort of 731 days, 95% CI 531–931; median OS in older cohort of 507 days (95% CI 288–726; *p* = 0.139; HR 1.367, 95% CI 0.902–2.072; Fig. 1a). However, further analyses with separating patients according to initial metastatic status indicate, that the younger cohort had a significant longer OS compared to the older cohort in an initial non-metastatic setting (median OS in young patients of 1226 days, 95% CI 703–1749; median OS in older patients of 801 days, 95% CI 267–1335; *p* = 0.028; HR 1.954, 95% CI 1.065–3.584; Fig. 1b). The initial metastatic cohorts had a similar overall survival (median OS in young patients of 343 days, 95% CI 166–520; median OS in older patients of 351 days, 95% CI 124–578; *p* = 0.931; HR 0.975, 95% CI 0.542–1.752; Fig. 1c).

Furthermore, there was a statistically significant difference between the overall survival of young and old patients with squamous cell carcinomas (median OS of young patients with SCC not measurable; median OS of older patients with SCC 390 days; 95% CI 122–658; *p* = 0.029), but not between adenocarcinomas (*p* = 0.484).

There was also a statistically significant difference between young and older patients concerning the extent of the metastatic disease. Young patients had a statistical significantly poorer OS in a metastatic setting with 2 or more metastatic sites (median OS in younger patients of 450 days, 95% CI 202–698; median OS in older patients of 646 days, 95% CI 0–2068; *p* = 0.033; HR 0.306, 95% CI 0.098–0.957; Fig. 1d).

There was no statistically significant difference between young and old patients when separated by gender (male *p* = 0.491, HR 1.196, 95% CI 0.718–1.994; female *p* = 0.123, HR 1.763, 95% CI 0.849–3.661), tumour location (oesophagus *p* = 0.263, HR 1.891, 95% CI 0.609–5.873; GEJ *p* = 0.872, HR 0.936, 95% CI 0.418–2.095; stomach *p* = 0.248, HR 1.380, 95% CI 0.797–2.388), Helicobacter pylori infection (negative *p* = 0.224, HR 1.497, 95% CI 0.778–2.880; positive *p* = 0.235, HR 2.053, 95% CI 0.611–6.890), nicotine (no consumption *p* = 0.063, HR 2.031, 95% CI 0.951–4.337; abuse *p* = 0.791, HR 0.910, 95% CI 0.452–1.830), grading (grade II *p* = 0.156, HR 1.844, 95% CI 0.782–4.347; grade III *p* = 0.077, HR 1.624, 95% CI 0.945–2.791), stage (stage I *p* = 0.538, HR 0.564, 95% CI 0.089–3.568; stage II *p* = 0.077, HR 2.847, 95% CI 0.850–9.534; stage III *p* = 0.195, HR 1.755, 95% CI 0.742–4.153; stage IV *p* = 0.960, HR 0.985, 95% CI 0.552–1.758) and Her 2 status (negative *p* = 0.230, HR 1.503, 95% CI 0.770–2.937; positive *p* = 0.493, HR 2.000, 95% CI 0.266–15.028).

#### One-, three- and five-year overall survival rates

50 (86%) and 48 (82%) patients survived more than one year in young and old patients’ groups, respectively (*p* = 0.798). 3- and 5-year survival was also similar in both groups with 20 (35%) and 10 (17%) and 18 (31%) and 8 (14%) patients, respectively (*p* = 0.843 and 0.798, respectively).

#### Overall survival in the older cohort

Within the older cohort only initial metastatic disease (no initial metastasis with a median OS of 801 days, 95% CI 267–1335; initial metastasis with a median OS of 351 days, 95% CI 124–578; *p* = 0.012, HR 2.064, 95% CI 1.157–3.684) and Her 2 status (unknown Her2 status with a median OS of 637 days, 95% CI 439–835; negative Her2 status with a median OS of 401 days, 95% CI 121–681; positive Her2 status with a median OS of 115 days; *p* = 0.049, HR 0.663, 95% CI 0.144–3.044) were associated statistical significantly with the OS, whereas age (*p* = 0.407, HR 1.020, 95% CI 0.973–1.070), gender (*p* = 0.174, HR 1.513, 95% CI 0.829–2.764), tumour location (*p* = 0.193, HR 1.335, 95% CI 0.881–2.022), histology (*p* = 0.766, HR 1.140, 95% CI 0.482–2.693), grading (*p* = 0.196, HR 1.061, 95% CI 0.759–1.481), stage (*p* = 0.088, HR 1.455, 95% CI 1.065–1.988), helicobacter pylori infection (*p* = 0.131, HR 1.259, 95% CI 0.550–2.883) as well as nicotine (*p* = 0.087, HR 0.419, 95% CI 0.189–0.929) were not.

#### Overall survival in the young cohort

Within the young cohort the parameters histology (adenocarcinoma with a median OS of 691 days, 95% CI 475–907; squamous cell carcinoma not measurable; *p* = 0.036, HR 0.244, 95% CI 0.059–1.019), initial metastatic disease (no initial metastasis with a median OS of 1226 days, 95% CI 703–1749; initial metastasis with a median OS of 90 days, 95% CI 165–520; *p* < 0.001, HR 4.057, 95% CI 2.110–7.800), grading (unknown grade with a median OS of 206 days, 95% CI 175–237; grade II with a median OS of 1279 days, 95% CI 134–2423; grade III with a median OS of 658 days, 95% CI 429–887; *p* = 0.005, HR 0.789, 95% CI 0.568–1.095) and stage (median OS of stage I 731 days; stage II 3446 days; stage III 1226 days; stage IV 343 days; p ≤ 0.001, HR 2.042, 95% CI 1.364–3.056) were also associated statistical significantly with the OS, whereas the parameters age (*p* = 0.567, HR 1.019, 95% CI 0.954–1.089), gender (*p* = 0.991, HR 1.004, 95% CI 0.527–1.914), tumour location (*p* = 0.277, HR 1.328, 95% CI 0.881–2.002), Helicobacter pylori infection (*p* = 0.09, HR 0.874, 95% CI 0.289–2.640), Her 2 status (*p* = 0.18, HR 0.422, 95% CI 0.121–1.473) and nicotine (*p* = 0.973, HR 1.070, 95% CI 0.524–2.183) were not.

### Tumour marker concentrations

Serum concentrations of CEA and CA19-9 were available in 39 patients before initiation of any kind of anti-tumour treatment and 6 months after the first established treatment. Concentrations of CEA and CA19-9 did not significantly change after initiation of the therapy in both groups (young patients, CEA-pre 72 µg/L versus CEA-post 26 µg/L, *p* = 0.2; young patients CA19-9-pre 1013 kU/L versus CA19-9-post 727 kU/L, *p* = 0.6; old patients, CEA-pre 144 µg/L versus CEA-post 300 µg/L, *p* = 0.2; old patients CA19-9-pre 181 kU/L versus CA 19-9-post 251 kU/L, *p* = 0.6). Interestingly, although not fulfilling the significance criteria, older patients tended to show increasing tumour marker levels after anti-tumour therapy, whereas the concentration of the tumour markers were decreasing among younger patients. Among these patients, cox regression analysis did not reveal any association of pre- or post-treatment concentrations of CEA or CA19-9 with the survival.

## Discussion

This study presents incidences of young patients with gastroesophageal tumours (GET) who are treated at the Department of Oncology, Medical University of Vienna and compares demographic, clinical and pathological data as well as outcomes of older GET patients who are treated at the same clinic. The definition of “young” patients varies among literature, where different cut-off values have been identified (Chaytors 1985; Dhobi et al. 2013; Kath et al. 2000; Lee et al. 2016; Seker et al. 2013). However, there is no official or specific definition by a recognised cancer organisation, such as the European Society for Medical Oncology, concerning the age limit of young patients with gastroesophageal cancer. In the frame of this study, we investigated patients whose age was under 45 years at the initial onset of a GET including oesophagus, gastroesophageal junction and stomach. This age limit was chosen according to current literature (Braga-Neto et al. 2018; Karrit et al. 2018; Yang et al. 2011).

### Comparison of young patients with the overall cohort

The portion of young patients was reported to be between 2 and 15% among all GET patients (Carvalho et al. 2004; Llanos et al. 2006; Santoro et al. 2007). In line with these reports, we found 7% of the young patients in our cohort of total 789 patients with a GET. Data on the clinical characteristics and outcome of young GET patients when compared to older patients was varying and even contradictive among previous reports. For instance, many studies found that these patients present a more aggressive disease stadium at the initial onset whereas some studies did not find any difference (Nakamura et al. 1999; Ramos-De la Medina et al. 2004). Among this current cohort, the proportion of young patients with stage IV disease seemed to be higher than the other population without any statistical significance. The ratio of female patients was higher among younger GET patients, which was associated with bad prognoses most probably due to the active sex hormone status (Kim et al. 2005; Liu et al. 2016; Wang et al. 2016; Zhou et al. 2016). Again, our cohort observed a tendency of higher numbers of female patients in the young GET group, which, however, did fail to reach statistical significance. Interestingly, our cohort found statistically higher amounts of stomach cancer in younger patients, whereas the rest of the cohort composed of more oesophagus and gastro-oesophageal junction tumours. This might be due to the long years of deformation of the reflux disease (Smyth et al. 2016), which might induce a malignant transformation of the gastroesophageal junction and oesophagus resulting in higher proportion of these locations in older ages.

The proportion of patients, whose diagnoses were made under 30 years was 10% of the young population. Unfortunately, little information was available on the family background of the patients; therefore, we cannot assess how hereditary factors participated in the development of cancer in this very young group.

### Comparison of characteristics of young versus older patients

As a next step, comparison of 58 patients, whose ages were 45 years and below, was done with matched older patients with GET. Some previous studies suggested poor differentiation of the tumour among young individuals (Kong et al. 2012; Wang et al. 2016), where we saw a tendency among this current cohort, however, without statistical significance. Helicobacter pylori infection was associated as one of the oncogenic processes in gastric cancer and was seen frequently in young patients when compared to older among the previous literature (Hirahashi et al. 2007). Our cohort, however, did not see any difference regarding to Helicobacter pylori infection of the tumour between young and old upper-GI patients. However, the status of Helicobacter pylori infection was associated with the overall survival in the combined cohort, which indicates that the infection itself might influence the overall survival independent from the age of the patient.

Although staging and grading between older and younger patients did not differ significantly, patients with younger ages showed higher numbers of metastasis at the metastatic setting and this associated with worse outcomes, when compared to older patients (Fig. 1d).

### Comparison of the overall survival in young versus older patients

In a non-metastatic setting younger patients showed a statistically significant longer overall survival than older patients (Fig. 1b). But concerning the extensive metastatic diseases with 2 or more metastatic sites the overall survival of young patients was statistical significantly poorer (Fig. 1d).

These findings might have implications for both aspects of the younger and older patients. From the aspect of the older patients, similar and even better survival times comparing to younger patients indicate that older patients might benefit from a multidisciplinary treatment of the GET. The advanced age of the patients is frequently considered as a limiting factor for clinicians for the treatment decisions in cases of aggressive anti-cancer treatment (Matthaiou and Papamichael 2017; Pak and Wang 2017). In the current cohort, older patients received identical types of anticancer treatments including surgical resection, radiation therapy and chemotherapy when compared to younger patients. Even the median cycle number of the chemotherapy did not differ when compared to younger patients, who are mostly believed to tolerate chemotherapy well. Notably, also side effects reported by the patients were similar. These findings might help to clinicians making decisions on the anti-tumour treatment strategies of the older patients and might encourage them to offer this patient group the complete treatment concept.

From the aspect of the younger patients, we have three main statements to consider concerning the overall survival in this analysis. First, young patients in an extensive metastatic setting had a poorer overall survival than older patients. This observation might strengthen the hypothesis that gastric cancer in young adults is often more aggressive and therefore progresses faster than in older patients (Lai et al. 2008; Saito et al. 2012). If this statement holds true, possible consequences might include broader screening methods as well as early supportive treatment arrangements such as psycho-oncological support and palliative care facilities for younger patients.

Second, young patients with a non-metastatic disease had a better overall survival than older patients in this analysis. This might be due to the facts, that younger people in general have a longer life expectancy than older ones and that younger patients have fewer comorbidities and are therefore in a better general condition. It is important to mention that in an initial non-metastatic setting the treatment goal is to cure the patient from the malignant disease by removing the primary tumour. Young patients are less likely to suffer from post-operative complications, thus might benefitting the tendency to a longer survival additionally (Liu et al. 2019).

Third, in this analysis younger patients with squamous cell carcinomas, which occur mostly in the oesophagus, had a longer overall survival compared to older patients. This again might be due to the fact, that older patients with oesophageal cancer have more comorbidities and therefore a poorer overall survival. However, as only six patients in the young cohort had squamous cell carcinomas, this result should be further investigated in a larger cohort.

A meta-analysis, which predominantly investigated patients from Japan and Korea demonstrated an improved outcome of younger patients when compared to older ones (Kong et al. 2012). It is important to mention that besides of the ethnic and socioeconomic factors, these countries include individuals in younger ages for the national-based screening programmes for gastric cancers, which might explain this finding of favourable outcomes (Liu et al. 2016; Zhou et al. 2016). Usually, younger patients are not included in preventive investigations, and even when those patients present symptoms such as stomach pain or reflux to the general practitioner, the symptoms are mostly not associated with a potential oncological disease. This subsequently often leads to the diagnosis of the tumour often in later stages, which obviously results in comparable or even worse prognosis with older patients. From the patients’ and clinicians’ points of view, more awareness of the symptoms at even younger ages and even broader screening might help with early diagnosis of these tumours. Especially in families with history of gastroesophageal tumours, endoscopy should be offered as a potential screening method. Thus, leading to more young patients being diagnosed in a non-metastatic setting, which might benefit the overall survival of these patients when compared to older ones.

Furthermore, if upper GI endoscopy was offered to the general population as a screening method, this might also lead to the avoidance of long-term Helicobacter pylori infections and a better surveillance of Barrett´s oesophagus. Thereby, potentially preventing the development of gastroesophageal cancer in the first place. Hence, endoscopy as a screening method might benefit not only young patients but the general population. However, large population based prospective screening studies are needed particularly in countries, where gastroesophageal cancer has a low prevalence, in order to make certain recommendations to the relatives of the patients and for the general population.

### Comparison of tumour markers in younger and older patients

In cases of early diagnosis of tumour diseases, the measurement of the circulating tumour markers plays a very promising role. Although CEA and CA-19-9 are not standardized tumour markers in gastroesophageal cancer, their sensitivity for gastric cancer is surmised to be greater than any other tumour markers’ (Yu et al. 2016). Furthermore, there is growing evidence, that a longitudinal analysis of circulating tumour markers might give evidence on the tumour burden and prognosis of these patients, thus particularly CEA and CA-19-9 are part of standard management in many countries (Lin et al. 2020). Serum concentrations of CEA and CA19-9 were available in some patients before and six months after the initiation of the first anti-tumour therapy. Although there were no associations of the circulating tumour markers in pre- and post-therapy samples, younger patients seemed to have decreasing levels, whereas older patients had increasing levels of tumour markers after the initiation of the therapy. Due to the size of this cohort, no clear conclusion of this finding can be reached; however, this observation should be investigated in larger prospective cohorts, which might lead to establishing different cut-off values between younger and older patients.

### Strengths and limitations of this study

Strengths and limitations of the study need to be considered. The patient population was homogenous and the younger cohort was matched to the older cohort. All patients were treated according to the individual decision of an interdisciplinary tumour board, which ensured the best possible treatment according to the respective standard of knowledge at the time of diagnosis. All patients were followed-up regularly.

One important limitation of this study, as it is a retrospective analysis, is missing data concerning the Her2 status, Helicobacter pylori status, grading, nicotine consumption and family history. Since history taking is obligatory and standardized at the General Hospital Vienna, most results were retrievable from the medical records. However, there were still missing parameters in some patients. Data was missing in 67 (32 young) patients concerning Her2 status, in 47 (26 young) patients concerning Helicobacter pylori status, in 13 (7 young) patients concerning grading and in 38 (17 young) patients concerning nicotine consumption.

Concerning the OS, 91 of the patients (78%, 42 patients in the young cohort) were already dead at the time of this analysis. The other patients were either still alive or lost to follow-up.

Although 796 patients with sufficient hospital data compose a large European cohort with upper gastrointestinal cancer, the matched cases were only 58 patients per group. This rather small sample size has to be considered when interpreting the obtained data.

Thus, to confirm the results of this study, a prospective study should be conducted to minimize missing data points.

### Conclusion

In conclusion, this retrospective survey demonstrates clinical characteristics of young patients with GET and describes a tendency of relative unfavourable outcomes in a metastatic setting and favourable outcomes in a non-metastatic setting for these patients when compared to older ones. Older patients within this group tolerated the anti-tumour treatment regimen in the same way as the younger patients and had comparable outcomes, which makes this group of patients potential candidates for the full programme of the anti-tumour treatments in metastatic settings. Furthermore, these retrospective findings again underline the importance of the early diagnosis of gastroesophageal cancer in young patients to possibly find more favourable disease conditions which might have an impact on the outcome.

## Abbreviations

CA19-9: Carbohydrate antigen 19-9
CEA: Carcinoembryonic antigen
CI: Confidence interval
FISH: Fluorescence in-situ hybridization
GI: Gastrointestinal
Her2: Human epidermal growth factor receptor 2
OS: Overall survival
SCC: Squamous cell carcinoma

## References

[CR1] Anderson WF, Camargo MC, Fraumeni JF, Correa P, Rosenberg PS, Rabkin CS (2010). Age-specific trends in incidence of noncardia gastric cancer in US adults. JAMA.

[CR2] Braga-Neto MB (2018). Clinical characteristics of distal gastric cancer in young adults from Northeastern Brazil. BMC Cancer.

[CR3] Carvalho R (2004). Early-onset gastric carcinomas display molecular characteristics distinct from gastric carcinomas occurring at a later age. J Pathol.

[CR4] Chaytors RG (1985). Gastric cancer in young people. Can Fam Phys.

[CR5] Dhobi MA (2013). Gastric cancer in young patients Int. J Surg Oncol.

[CR6] Eisenhauer EA (2009). New response evaluation criteria in solid tumours: revised RECIST guideline (version 1.1). Eur J Cancer.

[CR7] Hirahashi M, Yao T, Matsumoto T, Nishiyama K, Oya M, Iida M, Tsuneyoshi M (2007). Intramucosal gastric adenocarcinoma of poorly differentiated type in the young is characterized by Helicobacter pylori infection and antral lymphoid hyperplasia. Mod Pathol.

[CR8] Hofmann M (2008). Assessment of a HER2 scoring system for gastric cancer: results from a validation study. Histopathology.

[CR9] Karrit S (2018). Gastric cancer in young patients under the age of 45 years old: a comparative study with older patients. Ann Oncol.

[CR10] Kath R, Fiehler J, Schneider CP, Hoffken K (2000). Gastric cancer in very young adults: apropos four patients and a review of the literature. J Cancer Res Clin Oncol.

[CR11] Kim DY, Joo JK, Ryu SY, Park YK, Kim YJ, Kim SK (2005). Clinicopathologic characteristics of gastric carcinoma in elderly patients: a comparison with young patients. World J Gastroenterol.

[CR12] Kokkola A, Sipponen P (2001). Gastric carcinoma in young adults. Hepatogastroenterology.

[CR13] Kong X, Wang JL, Chen HM, Fang JY (2012). Comparison of the clinicopathological characteristics of young and elderly patients with gastric carcinoma: a meta analysis. J Surg Oncol.

[CR14] Lai JF (2008). Clinicopathologic characteristics and prognosis for young gastric adenocarcinoma patients after curative resection. Ann Surg Oncol.

[CR15] Lee J, Lee MA, Kim I-H, Roh S-Y (2016). Clinical characteristics of young-age onset gastric cancer in Korea. BMC Gastroenterol.

[CR16] Lin J-P (2020). Prognostic significance of pre- and post-operative tumour markers for patients with gastric cancer. Br J Cancer.

[CR17] Liu S (2016). Clinicopathological features and prognosis of gastric cancer in young patients. BMC Cancer.

[CR18] Liu W, Quan H, Chen X, Ouyang Y, Xiao H (2019). Clinicopathological features and prognosis of young gastric cancer patients following radical gastrectomy: a propensity score matching analysis. Sci Rep.

[CR19] Llanos O, Butte JM, Crovari F, Duarte I, Guzman S (2006). Survival of young patients after gastrectomy for gastric cancer. World J Surg.

[CR20] Matthaiou C, Papamichael D (2017). Management of gastric cancer in older adults. J Geriatric Oncol.

[CR21] Nakamura T, Yao T, Niho Y, Tsuneyoshi M (1999). A clinicopathological study in young patients with gastric carcinoma. J Surg Oncol.

[CR22] Pak LM, Wang J (2017). The appropriate treatment for elderly gastric cancer patients. Art Surg.

[CR23] Ramos-De la Medina A, Salgado-Nesme N, Torres-Villalobos G, Medina-Franco H (2004). Clinicopathologic characteristics of gastric cancer in a young patient population. J Gastrointest Surg.

[CR24] Saito H, Takaya S, Fukumoto Y, Osaki T, Tatebe S, Ikeguchi M (2012). Clinicopathologic characteristics and prognosis of gastric cancer in young patients Yonago. Acta Med.

[CR25] Santoro R, Carboni F, Lepiane P, Ettorre GM, Santoro E (2007). Clinicopathological features and prognosis of gastric cancer in young European adults. Br J Surg.

[CR26] Seker M, Aksoy S, Ozdemir NY, Uncu D, Zengin N (2013). Clinicopathologic features of gastric cancer in young patients Saudi. J Gastroenterol.

[CR27] Smith BR, Stabile BE (2009). Extreme aggressiveness and lethality of gastric adenocarcinoma in the very young. Arch Surg.

[CR28] Smyth EC, Verheij M, Allum W, Cunningham D, Cervantes A, Arnold D, Committee EG (2016) Gastric cancer: ESMO clinical practice guidelines for diagnosis, treatment and follow-up. Ann Oncol 27:v38–v49. 10.1093/annonc/mdw350 10.1093/annonc/mdw350 27664260

[CR29] Takatsu Y (2016). Clinicopathological features of gastric cancer in young patients. Gastric Cancer.

[CR30] Theuer CP (1996). Gastric adenocarcinoma in patients 40 years of age or younger. Am J Surg.

[CR31] Van Cutsem E, Sagaert X, Topal B, Haustermans K, Prenen H (2016). Gastric cancer. Lancet.

[CR32] Wang Z, Xu J, Shi Z, Shen X, Luo T, Bi J, Nie M (2016). Clinicopathologic characteristics and prognostic of gastric cancer in young patients. Scand J Gastroenterol.

[CR33] Yang D (2011). Survival of metastatic gastric cancer: Significance of age, sex and race/ethnicity. J Gastrointest Oncol.

[CR34] Yu J, Zhang S, Zhao B (2016). Differences and correlation of serum CEA, CA19–9 and CA72–4 in gastric cancer. Mol Clin Oncol.

[CR35] Zhou F, Shi J, Fang C, Zou X, Huang Q (2016). Gastric Carcinomas in Young (Younger than 40 Years) Chinese Patients: Clinicopathology, Family History, and Postresection Survival. Medicine.

